# Histological Examination of Retrieved ePTFE Membranes Following Regenerative Surgery of Intrabony Defects Treated with Platelet-rich Plasma and Bone Substitutes

**DOI:** 10.3290/j.ohpd.b2805491

**Published:** 2022-03-14

**Authors:** Ferenc Dőri, Anton Sculean, Dániel Takács, Zsuzsanna Suba

**Affiliations:** a Professor, Department of Periodontology, Semmelweis University, Budapest, Hungary. Study design, treatment of patients, wrote the manuscript.; b Professor and Chair, Department of Periodontology, University of Bern, Bern, Switzerland. Study design, proofread the manuscript.; c PhD Student, Department of Oral- and Maxillofacial Surgery, Semmelweis University, Budapest. Recording clinical data, statistical analysis, proofread the manuscript.; d Professor, Department of Molecular Pathology, National Institute of Oncology, Budapest, Hungary. Study design, histological evaluation, wrote the manuscript.

**Keywords:** β-tricalcium phosphate, guided tissue regeneration, histological evaluation, natural bone mineral, platelet-rich plasma

## Abstract

**Purpose:**

Regenerative periodontal therapy using platelet-rich plasma (PRP) and bone substitutes with guided tissue regeneration (GTR) have been proposed as a therapeutic method to enhance the outcome of regenerative surgery. This includes light microscopic evaluation of retrieved ePTFE membranes to assess formation of new connective tissue attachment, and following the regeneration process. The objectives of this study were to evaluate the histological findings of retrieved ePTFE membranes using PRP and bone substitutes, the effect of PRP on graft materials, and the correlation of the findings with the clinical outcomes.

**Materials and Methods:**

Seventy-two (72) patients with chronic periodontitis, each of whom had one deep intrabony defect, were randomly included in two studies and treated using the same type of membrane and different bone substitutes. In the first study, 17 cases were treated with a natural bone mineral and a non-resorbable membrane (NBM + GTR), and 17 cases were treated with PRP + NBM + GTR. In the second study, 19 patients were treated with β-tricalcium phosphate and a non-resorbable membrane (β-TCP + GTR), and 19 patients were treated with PRP + β-TCP + GTR. In both studies, tissue integration of the retrieved ePTFE membranes and attached remnants were evaluated histologically.

**Results:**

Histological scores showed that membranes combined with NBM are better integrated than membranes combined with β-TCP; the difference between the two decreased with the addition of PRP. The application of PRP had no significant effect on the quality of membrane integration combined with NBM, whilst significantly improving the integration quality when combined with β-TCP. No correlations were detected between the histological scores and the clinical attachment level (CAL) gain in any of the groups.

**Conclusions:**

The present results indicate that: a) application of β-TCP and PRP may enhance membrane integration and periodontal healing, and b) histological examination of retrieved membranes may provide valuable additional information with regard to the clinical findings.

Periodontal regeneration and formation of new attachment structures can be stimulated by special conditions.^4,15,28^ Early methods to promote the formation of connective tissue or to fill osseous defects did not result in adequate regeneration of the natural periodontal tissues.^19,30^ Without a proper method which allows selective cellular repopulation of the root surface, the supporting system of the bone, i.e. the periodontal ligament and the cementum, can only regenerate unpredictably. It has long been established that new cementum and periodontal ligament cells can develop on the cleaned root surface if they are isolated from other tissues during the healing period.^14,24,25^ This isolation during initial healing allows the reestablishment of the precursors of the supporting tissues.

The first clinically applicable device designed for guided tissue regeneration (GTR) was the expanded polytetrafluoroethylene (ePTFE) barrier, a non-resorbable membrane, which facilitates the ingrowth of connective tissue and prevents the apical migration of the epithelium in the early phase of healing.^16^ Light microscopic evaluation of retrieved ePTFE membranes is a useful method to assess both their biocompatibility and their sealing capacity.^27^ Analysis using scanning electron microscopy (SEM) demonstrated that retrieval of non-resorbable membranes can be useful to evaluate the formation of new connective tissue, follow the regenerative process, and assess the subsequent success or failure of periodontal regeneration.^2,37^ Application of certain growth factors has resulted in earlier bone regeneration and more mature bone production, even in cases of bone grafting.^23^

The most frequently studied natural source of the growth factors is platelet-rich plasma (PRP). Platelet-derived growth factors can bind to the endothelial cells and initiate the ingrowth of capillaries,^33^ thus providing sufficient vascularisation, which is necessary for osteogenesis. Furthermore, platelets can release transforming growth factors (TGF-β1, TGF-β2), which can bind to the endosteal osteoblasts and marrow stem cells to initiate their proliferation.^23^

Although various regenerative therapies influence the healing process, clinical studies on the role of PRP in periodontal healing often result in contradictory conclusions:^6,10,17,29,31,34,45^ whilst combination of PRP and bone substitutes can lead to significant clinical improvement in deep intrabony periodontal defects,^17,46^ other studies have found that PRP did not significantly augment the effects of GTR.^5,11^

Using light microscopy to verify the additive bone-forming capacity of PRP when examining preprosthetic sinus floor elevation provides an excellent possibility for quantitative histological evaluation of new bone formation enhanced by bone replacement materials.^40^ Cylindrical biopsy specimens can be taken from the augmented, regenerating bone before the insertion of artificial roots. Histological results of such studies confirmed that PRP addition to bone substitute materials may enhance the activity of bone regeneration, especially in the early phase.^18,40^ Unfortunately, the methods used for histological analysis in bone augmentation surgery combined with implantation are not available in periodontal regenerative surgery; however, light microscopy and SEM analysis of non-resorbable membranes can provide data on bone grafts and biological mediators used in periodontal surgery.

The objective of this study was to histologically evaluate the tissue/membrane interfaces of the retrieved ePTFE membranes used in natural bone mineral (NBM) or β-tricalcium phosphate (β-TCP) treatments of intrabony defects, applied with or without platelet-rich plasma (PRP), and to assess the influence of PRP on membrane integration and healing in patients with chronic periodontitis. A new, semiquantitative histological scoring system was introduced to correctly evaluate and compare membranes retrieved from variously treated periodontal bone defects. A further aim was to analyse the contingent relation between the semi-quantitatively established histological scores and the clinical attachment level (CAL) gain, with or without PRP.

## Materials and Methods

### Patient Population and Clinical Procedures

Seventy-two patients (42 females and 30 males), each of whom exhibited at least one deep intrabony defect (probing depth, PD: 8-10 mm), were treated surgically after debridement and successful oral hygiene instructions and motivation. All subjects were non-smokers and in good systemic health with no contraindications for periodontal or oral surgery. The study was conducted at the Department of Periodontology, Semmelweis University. The study protocol was approved by the ethics board of Semmelweis University, Budapest, and was in accordance with the Helsinki Declaration of 1975, as revised in 2000.

Two studies were performed in parallel. In the first study (Study I), 17 patients were treated with a natural bone mineral (Bio-Oss, Geistlich; Wolhusen, Switzerland) and a non-resorbable membrane (Gore-Tex Periodontal Material, W.L. Gore; Flagstaff, AZ, USA) (NBM + GTR), and 17 cases were treated with PRP + NBM + GTR. In the second study (Study II), 19 patients were treated with β-tricalcium phosphate (Cerasorb, Curasan Pharma; Kleinostheim, Germany) and a non-resorbable membrane (β-TCP + GTR), and 19 patients were treated with PRP + β-TCP + GTR. The main clinical parameter of the study was the clinical attachment level (CAL).

### Randomisation

In each study, prior to the surgery, the defects were assigned to the two treatment groups using a randomised block approach, in which prognostic variables, INTRA (the distance from the alveolar bone crest to the bottom of the defect) and CAL, were used to decrease outcome variability. INTRA was estimated before surgery based on radiographs and transgingival bone sounding recordings.

### Intraexaminer Reproducibility

Five subjects, each having 10 teeth (single- and multi-rooted) with PD > 6 mm on at least one aspect, were used to calibrate the examiner. The examiner evaluated the subjects on two occasions, 48 h apart. Calibration was accepted if > 90% of the recordings could be reproduced within a 1.0-mm difference.

### Surgical Procedure, PRP Preparation

All surgeries were performed by the same experienced operator (FD). Following local anaesthesia, intracrevicular incisions were made, and full-thickness mucoperiosteal flaps were raised vestibularly and orally. The granulation tissue was removed from the defects, and the roots were scaled and planed thoroughly using hand and ultrasonic instruments. No conditioning of the root surfaces was performed. In the test groups, the bone substitutes were mixed with PRP. Following grafting, the non-bioresorbable expanded polytetrafluoroethylene (ePTFE) membrane was trimmed and adapted over the entire defect to achieve a 2- to 3-mm coverage of the surrounding alveolar bone and to ensure stability of the wound and the graft material. The membranes were fixed to the same and/or neighbouring teeth with sling sutures. The same surgical protocol was used for control groups, but without PRP in the bone substitute. Finally, the flaps were repositioned coronally and closed with vertical or horizontal mattress sutures.

PRP preparation was performed by using a standardised kit (Curasan PRP kit) immediately prior to operation. It has been demonstrated that PRP volumes prepared with this technique contain a mean platelet count of 2520 ± 834 x 10^3^/μl and high mean concentrations of growth factors (ie, 295 ng/μl PDGF-AB and 500 ng/μl TGF-β1).^3^

### Postoperative Care

Postoperative care consisted of a 0.2% chlorhexidine digluconate solution mouth rinse twice a day over the first 2–3 postoperative weeks. After this period, tooth brushing was resumed in the operated areas. All the subjects took 3 x 625 mg Augmentin (GlaxoSmithKline; Brentford, Middlesex, UK) for 7 days postoperatively. Flap sutures were removed after 10–12 days. After the surgery, weekly clinical checks were performed until membrane removal.

### Membrane Removal

**Fig 1:**
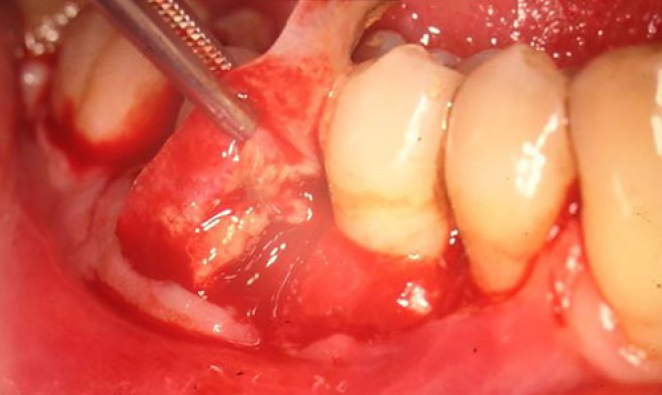
Membrane removal.

After 6 weeks, performing partial thickness flap exposure (Fig 1), the ePTFE membranes were surgically removed under local anaesthesia. In an attempt to remove the epithelium, the inner surface of the flap was gently curetted, and using interproximal sutures, the flap was sutured to the surface as close to the cementoenamel junction as possible. Hygiene instructions were given again, and the sutures were removed after 5-6 days.

### Histological Procedure

Retrieved ePTFE membranes were subjected to histological analysis. The membranes and the tissue remnants attached to the membranes were fixed immediately in a 4% buffered formaldehyde solution for one day. After fixation, a series of 1-mm-wide mesio-distal strips were cut from these membranes using a razor blade. The tissue samples were decalcified, dehydrated, and embedded into paraffin. Slides were stained with haematoxylin and eosin, toluidin blue, and using Goldner’s trichrome method.

### Histological Evaluation

**Fig 2:**
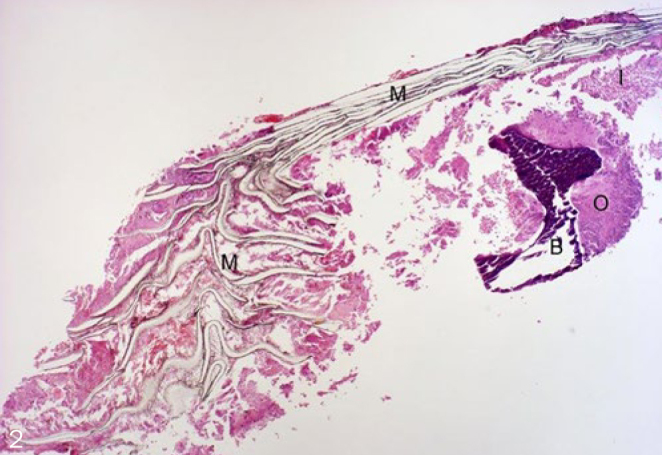
Score 1 (4X magnification, haematoxylin-eosin, H-E). M: membrane; B: bone; O: osteoid tissue; I: infiltrate.

**Fig 3:**
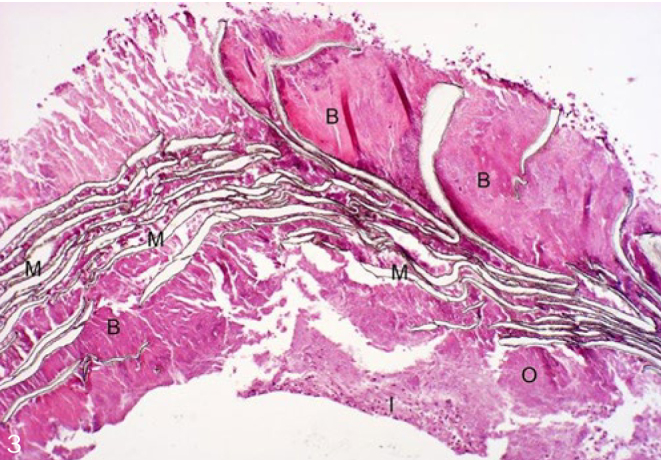
NBM+GTR (10X magnification, H-E). M: membrane; B: bone; O: osteoid tissue; I: infiltrate.

**Fig 4:**
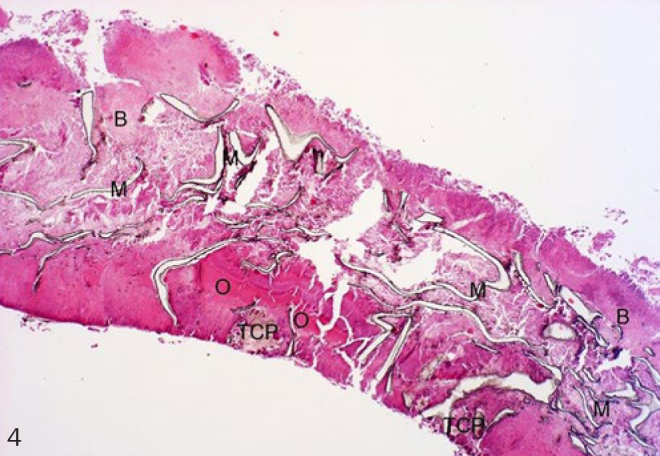
β-TCP+GTR (4X, H-E). M: membrane; B: bone; O: osteoid tissue; TCP: tricalcium phosphate.

**Fig 5:**
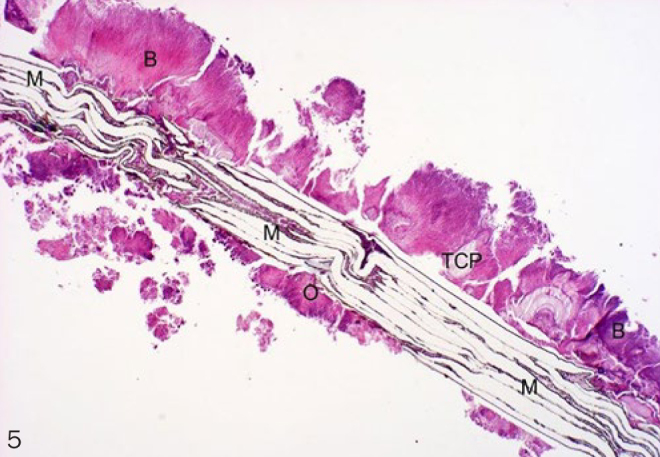
Score 2 (10X magnification, H-E). M: membrane; B: bone; O: osteoid tissue; TCP: tricalcium phosphate.

**Fig 6:**
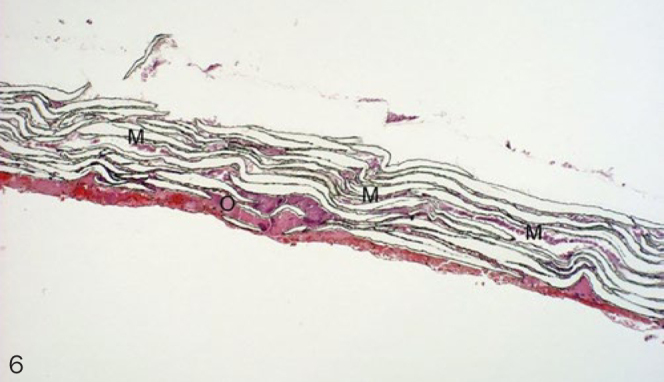
Score 3 (10X magnification, H-E). M: membrane; O: osteoid tissue.

Histological evaluation was performed under normally transmitted light. Integration of the membranes into the surrounding newly formed tissues and the cellular reactions on their surfaces were evaluated. Examined membrane samples were divided into three groups by scoring them according to their histological picture. Extensive lamellar disintegration of the membrane combined with hard tissue ingrowths in the cleavages give a score of 1 (Fig 2). In this group, hard tissue remnants were attached to both the outer and the inner surfaces of well-integrated membranes, and small islands of newly formed bone were observed (Figs 3 and 4). Retrieved membrane received a score of 2 if it showed moderate lamellar destruction, and exhibited only small nodules of osteoid or new bone tissue (Fig 5). Score 3 was assigned when the retrieved membrane was compact, without any sign of disintegration, and no hard tissue precursors were found attached to its surfaces (Fig 6). Presence of an inflammatory infiltrate along the surfaces and missing of the hard material were also included in this group. The histological scoring was performed independently by two pathologists.

In each study, the data from the two groups were compared to identify any differences in membrane incorporation when using bone substitute materials with or without PRP.

### Statistical Analysis

Mean score values of clinical measurements at baseline and at six months, and histological findings and standard deviations were calculated for each group with different periodontal treatment. After testing for normal distribution, the paired t-test (difference betweem baseline and 6 months within one group) and the unpaired t-test (difference between the two groups) were used to determine the levels of statistical significance. The chi-squared test was used to compare histological scores of retrieved e-PTFE membranes of the treatment groups after 6 weeks. Values of p < 0.05 were considered statistically significant. Given ≥ 1 mm as a statistically significant difference between the groups, the power of the study was calculated to be 0.70.

## Results

### Clinical Results

The clinical results are summarised in Tables 1 and 2. CAL improved statistically significantly compared to the baseline value in each group after six months. The difference between the CAL values of the two groups in each study was statistically insignificant. Using the same membrane and two different graft materials – a natural bone mineral and β-tricalcium phosphate – with and without platelet-rich plasma resulted in a distinct improvement of clinical parameters. The addition of PRP to bone substitutes did not affect the clinical healing. Detailed one-year clinical results are available in the earlier publications.^9,11^

**Table 1:** Study I: Mean CAL values at baseline and at six months (n = 17/17)

	Baseline	Six months	p-value
CAL (mm)			
NBM + GTR	9.7 ± 2.2	4.5 ± 2.0	p < 0.001
PRP + NBM + GTR	12.3 ± 2.4	4.8 ± 1.8	p < 0.001
p-value	NS	NS	

**Table 2:** Study II: Mean CAL values at baseline and at six months (n = 19/19)

	Baseline	Six months	p-value
CAL (mm)			
β-TCP + GTR	9.3 ± 2.3	4.5 ± 2.3	p < 0.001
PRP + β-TCP + GTR	11.0 ± 2.9	4.9 ± 2.2	p < 0.001
p-value	NS	NS	

### Scoring of ePTFE Membranes

Scoring of the tissue integration of the membranes was separately evaluated in the two studies. In the NBM + GTR group (n = 17), seven membranes had a score of 1, six membranes a score of 2, and four membranes with score 3. In the PRP + NBM + GTR group (n = 17), seven membranes received a score ofo 1, while five membranes were scored 2, and five scored 3. In the β-TCP + GTR group (n = 19), there was only one membrane which scored 1, eight cases which scored 2, and ten cases which scored 3. In the PRP + β-TCP + GTR group (n = 19), two membranes were scored 1, eleven cases scored 2, and six cases scored 3.

Based on histological scores, the membranes combined with NBM appeared to be better integrated, with a more efficient barrier function, than the ones with β-TCP. However, the difference in quality of membrane integration decreased when PRP was added to the graft materials in favour of β-TCP.

The application of PRP had no statistically significant effect on the quality of ePTFE membrane integration combined with NBM, whilst statistically significantly improving membrane integration in combination with β-TCP (Table 3).

**Table 3:** Histological evaluation of retrieved membranes after six weeks

	Histological scoring		Histological scoring	p-value
NBM + GTR	1.7 ± 0.7	PRP + NBM + GTR	1.8 ± 0.8	NS
β-TCP + GTR	2.5 ± 0.5	PRP + β-TCP + GTR	2.2 ± 0.5	p < 0.05

### Comparison of Clinical and Histological Results

Patients were grouped according to the histological membrane scores, and the resulting groups were analysed based on the percentage of CAL gain. Calculations of the percentages of CAL gain revealed a tendency for better clinical outcomes in the test group, but without reaching statistical significance in both studies. Following clinical assessment of the impact of ePTFE membrane integration after six months, it was established that no correlations were detected between histological scoring of the retrieved membranes and the achieved attachment gain in the differently treated groups (Tables 4 and 5).

**Table 4:** Study I: Mean CAL gain (%) values related to membrane scores (n = 17/17)

Membrane score	NBM + GTR CAL gain (%)	PRP + NBM + GTR CAL gain (%)	Mean CAL gain (%)
Score 1	60.85 (7/17)	67.28 (7/17)	64.06 (14/34)
Score 2	39.33 (6/17)	59.60 (5/17)	49.46 (11/34)
Score 3	58.50 (4/17)	52.40 (5/17)	55.45 (9/34)
	52.89 (17)	59.76 (17)	

**Table 5:** Study II: Mean CAL gain (%)related to membrane scores (n = 19/19)

Membrane score	β-TCP + GTR CAL gain (%)	PRP + β-TCP + GTR CAL gain (%)	Mean CAL gain (%)
Score 1	58.00 (1/19)	58.50 (2/19)	58.25 (3/38)
Score 2	45.50 (8/19)	54.09 (11/19)	49.79 (19/38)
Score 3	55.40 (10/19)	58.33 (6/19)	56.86 (16/38)
	52.96 (19)	56.97 (19)	

## Discussion

Several well-known as well as unknown factors can influence the predictability and success rate of periodontal regenerative therapy. Local factors associated with the postoperative results are: the dimensions and the morphology of osseous lesions, the surgical technique, the postoperative dental plaque control, and the possibility and extent of bacterial contamination.

The effectiveness of guided tissue regeneration has been reported in several publications. Clinically, long-term reports are providing important information regarding the efficacy of the regenerative method. The most important aspects to evaluate are long-term tooth retention, the recurrence of an inflammation, the maintenance of the clinical outcomes and the costs.^7,8^

Histological and microbiological analysis of retrieved membranes can provide data regarding the formation of new connective tissue, the presence of inflammatory cells and bacterial deposits, and their potential influence on periodontal healing and regeneration.^2,27,37^

Machtei et al^21^ histologically demonstrated that the number of fibroblasts on the inner surface of the membranes correlated with a reduction of defect dimensions. This finding suggests that histological analysis of the retrieved membranes might be a useful predictor of future regenerative changes.

Selvig et al^38^ reported predominantly fibroblast-like cells, observed in the mid- and the deep parts of the investigated ePTFE membrane surfaces. The inflammatory cells were associated with connective tissue structures as well as with bacterial deposits. Distribution of the adherent structures on the inner and outer surfaces of the membrane showed no differences.

An animal study revealed a higher degree of epithelial downgrowth following the use of ePTFE membranes and a low incidence of acute inflammatory reaction.^13^ On the ePTFE specimens, where PMN leukocytes were slightly more common: these were found in greatest density within the voids of the mesh.

An vitro studies^35^ showed that the low protein-binding capacity of the ePTFE material and the rough-textured surface of the barrier resulted in the inhibition of epithelial cell migration. Additional studies analysed various membranes and reported only a minimal amount of initial cell attachment to ePTFE material, thus suggesting that the growth rate of periodontal ligament cells was highly influenced by the material used.^12,41^ Minimal tissue integration may be an advantage for membrane retrieval; however, this may also create potential problems for membrane stability, and thus may interfere with wound healing.

Yoshinari et al^47^ observed a higher number of adherent and invading mononuclear inflammatory cells, present even in the mid-part of the ePTFE membranes, accompanied by bacterial contamination, which had a negative influence on clinical attachment gain.

Cellular and molecular events related to cell-populated retrieved membranes were also analysed. It seems that cells linked to the membrane not only possess regenerative ability, but also convey inflammatory signals.^44^ Furthermore, cells associated with the PTFE membranes from GTR procedures were found to express higher levels of the extracellular matrix proteins collagen type 1 and fibronectin.^20^ A recent review^26^ concluded that cellular and molecular activities in the membranes are linked to the promoted bone regeneration in the underlying defect and that incorporating growth factors and cells in membranes or with graft materials may augment the regenerative processes in the defects.

Notwithstanding the above, earlier studies revealed controversial results related to the bone regenerative potential of platelet-rich plasma. PRP showed low regenerative ability on autogenous sinus grafts in sheep.^18^ The periodontal regeneration process was evaluated in canine mandible using β-TCP alone or combined with PRP. Histomorphometric results suggested more intense bone regeneration in the early healing phase following the topical application of PRP, but 24 weeks after grafting, osteogenetic activity was nearly equal in the two groups.^39^

In our study, histological evaluation of the retrieved ePTFE membranes yielded important results: when combined with platelet-rich plasma, the bone-forming activity of β-tricalcium phosphate was facilitated 6 weeks after periodontal surgery. At the same time, the NBM activity was not statistically significantly enhanced by PRP application.

It has been shown that NBM may enhance periodontal regeneration.^36,43^ We assume that this observation can explain the present result that the application of PRP had no statistically significant effect on the quality of ePTFE membrane integration combined with NBM.

Other studies^22,34^ have shown that growth factors may enhance the regenerative effect of β-TCP. It is anticipated that the influence of PRP can cause a statistically significant improvement in membrane integration when combined with β-TCP.

Six-month clinical evaluations indicate parallel clinical outcomes in the case of the two grafts, with or without PRP. The advantages of PRP combined with β-TCP shown by histological scores disappeared during the 6-month period of periodontal healing. One-year clinical evaluation of these methods also showed statistically significant improvements in PD and CAL values; however, no statistically significant differences were seen between the two groups with and without PRP application.^9,11^ The present results demonstrate that, in clinical trials, histological examination of the retrieved membranes may also provide valuable information. Selvig et al^37^ reported large portions of membrane surface free from tissue deposits, suggesting their detachment during the re-entry procedure, so the ‘membrane integration score’ could underestimate the extent of connective tissue infiltration.^37^ This observation is one of the limitations in our study.

The present histological findings suggest that application of β-TCP and PRP complex can be beneficial in regenerative surgery of intrabony periodontal defects, although the data failed to reveal a close correlation between histological membrane scores and the clinical results. These differences require further clinical and histological studies to clarify the role of PRP in periodontal healing processes. To date, consensus is lacking on the role of platelet-rich plasma in wound healing and tissue regeneration in periodontology.^1,9,10,29,31,32,34,42,48^ To address this challenge, it may be necessary to consider various other factors, such as age, gender, metabolism, and hormonal factors, which can also affect biological processes involved in periodontal wound healing and regeneration.

## Conclusion

Histological results from this study indicate that the application of ß-TCP and PRP complex can be useful in periodontal regenerative surgery. Histological examination of the retrieved membranes may provide valuable information on the clinical outcomes.
